# Predictors of patient uptake of colorectal cancer gene environment risk assessment

**DOI:** 10.1186/gm393

**Published:** 2012-11-29

**Authors:** Michael J Hall, Sharon L Manne, Ronald E Myers, Eileen M Keenan, Andrew M Balshem, David S Weinberg

**Affiliations:** 1 Cancer Prevention and Control, Fox Chase Cancer Center, 333 Cottman Avenue, Philadelphia, PA 19038, USA; 2 Cancer Prevention and Control, Cancer Institute of New Jersey, 195 Little Albany Street, New Brunswick, NJ 08901, USA; 3 Department of Medical Oncology, Jefferson Medical College, 1025 Walnut Street, Philadelphia, PA 19107, USA

## Abstract

**Background:**

In an ongoing clinical trial, the genetic and environmental risk assessment (GERA) blood test offers subjects information about personal colorectal cancer risk through measurement of two novel low-to-moderate risk factors. We sought to examine predictors of uptake of the GERA blood test among participants randomized to the Intervention arm.

**Methods:**

Primary care patients aged 50 to 74 years eligible for colorectal cancer screening are randomized to receive a mailed stool blood test kit to complete at home (Control) or to the control condition plus an in-office blood test called GERA that includes assessment of red blood cell folate and DNA-testing for two *MTHFR *(methylenetetrahydrofolate reductase) single nucleotide polymorphisms (SNPs) (Intervention). For the present study, baseline survey data are examined in participants randomized to the Intervention.

**Results:**

The first 351 intervention participants (161 African American/190 white) were identified. Overall, 249 (70.9%) completed GERA testing. Predictors of GERA uptake included race (African American race, odds ratio (OR) 0.51 (0.29 to 0.87)), and being more knowledgeable about GERA and colorectal cancer screening (OR 1.09 (1.01 to 1.18)). Being married (OR 1.81 (1.09 to 3.00)) was also significant in the multivariable model.

**Conclusions:**

Participant uptake of GERA testing was high. GERA uptake varied, however, according to socio-demographic background and knowledge.

## Background

Colorectal cancer (CRC) is the third most common cancer of US men and women, with approximately 150,000 new diagnoses in 2012 [1]. While a small number of CRCs are known to be caused by mutations in high penetrance cancer genes such as those associated with familial adenomatous polyposis or Lynch syndrome, most cases of CRC appear to be sporadic, and likely arise from risks associated with both low penetrance genes and environmental risks such as dietary or toxin exposures. Colonoscopy screening in adults is proven to lower the risk of developing CRC and is endorsed by the US Preventive Services Task Force [2]. Despite recent increases in general US population screening, improvements are still needed. Screening rates among underserved populations continue to lag behind those of white Americans [3]. Improving CRC screening rates remains a national health care goal [2,3].

Experts have hypothesized that providing personalized genetic susceptibility feedback may serve as an important link between public health goals and individual motivation to engage in healthy behaviors such as cancer screening [4,5]. A number of SNPs associated with generally modest (5 to 20%) increases in cancer risk have been identified, and several studies have to date examined the impact of genetic susceptibility feedback on health behaviors either through hypothetical scenarios or through offering single-gene or so-called multiplex genetic testing [6-9]. In adult populations both at increased risk for cancer and unselected for cancer risk, interest in genetic susceptibility feedback is generally high, and experts have supported a potential for large impact on prevention behaviors [4,5]. A recent meta-analysis of the impact of genetic susceptibility feedback on smoking and physical activity outcomes, however, suggested limited effectiveness [10].

Clinical risk assessment for increased genetic risk of disease in adults currently consists primarily of intensive counseling and testing for mutations in highly penetrant risk genes. Interest in and uptake of novel forms of genetic susceptibility feedback testing, examined largely through research, remains variable, and has been shown to be moderated by a number of demographic, psychological, and psychosocial factors [6-8,11,12]. Little is currently known about how patients will react to a genetic susceptibility feedback test that examines a combination of genetic and environmental (for example, dietary) risk factors, and whether such a combination test might prove to be a superior stimulus for health behavior change compared to a genetic susceptibility feedback test alone. Research has demonstrated that the public assigns similar importance to genetic and behavioral sources of risk across a wide range of adult diseases, supporting at face value the appeal of gene-environment susceptibility testing [6,13,14]. Further, studies have suggested that African Americans (AAs; versus white) may have more positive attitudes towards genetic testing research in which a dual genetic/behavioral risk model of disease is emphasized [13,14].

The majority of studies that examine interest in and utilization of genetic testing have been performed among individuals with a strong personal or family history of disease who are eligible for predictive genetic testing (for example, *BRCA1/2*). Here, testing uptake has been associated with elevated perceived cancer risk, higher knowledge/awareness of genetic testing for cancer risk, and demographic factors including white race. Recent literature examining SNP-based genetic susceptibility feedback testing provides additional insight into reasons why individuals may or may not pursue genetic testing presented in a different context. For example, like patients seeking a predictive genetic testing for cancer risk (for example, *BRCA1/2*), individuals with elevated objective or perceived risk of adult disease may seek genetic susceptibility feedback due to increased perceived relevance of the testing to their health [6,7,9,12,15], increased cancer worry [6,8], or to support an already elevated perceived risk of developing cancer or motivation to change behavior, even when testing for a low penetrance risk gene [6,8,16]. These same high-risk individuals may also express strong intentions to modify behaviors dependent on further information about risk [7,8,11,15], but may ultimately fail to move forward with testing due to practical (for example, access) and psychological barriers (for example, low motivation or awareness) [11]. On the contrary, motivations for uptake of genetic susceptibility feedback testing in average risk groups may derive from other sources. Perceived relevance of genetic susceptibility testing for disease risk is likely to be lower in this group. Taking this into consideration, motivations for testing may be driven by psychological optimism toward genetic testing and genetics research, favorable views of knowledge enhancement afforded by testing, or to positively support currently unattained personal health goals [9]. In other instances, these individuals might also use a genetic susceptibility feedback result to defend a current adverse health behavior (for example, smoking, screening avoidance), or may be more apt to avoid testing due to distrust of test results or a desire to avoid negative information about a current adverse health behavior [9,14].

Moreover, among average risk groups, low objective knowledge of CRC risks, screening, or standard tests coupled with less experience with genetics and genetic testing may be central to attitudes and behaviors toward genetic susceptibility feedback testing. Multiple studies have shown that the general public lacks basic knowledge and understanding about genetics and genetic testing [17-20]. Comparatively lower knowledge and awareness of genetic tests for cancer risk has also been recorded among less educated individuals, and among minorities and underserved individuals relative to white Americans [17,18,21-25], particularly AAs [21,26-28]. Low knowledge of genetic testing has been negatively associated with testing interest and testing uptake in the literature examining predictive genetic testing [17,18,22-24]. In particular, studies have reported low knowledge of genetics associated with strong interest and/or intention to test but yet ultimately low completion of testing, particularly in underserved and less educated groups. Higher overall information needs and information seeking support needs in this group may contribute to the intention-uptake disconnect [6,14,29].

Our research group recently reported on the feasibility of offering a gene-environment risk assessment (GERA) study in which testing results for two markers of CRC risk, serum folate level and two SNPs in the *MTHFR *gene (methylentetrahydrofolate reductase), were made available to adults at average risk for CRC [30,31]. An ongoing randomized study is currently examining the impact of GERA testing on CRC screening compliance. In routine study monitoring, an unexplained lower rate of completion of GERA testing in subjects randomized to the intervention arm was detected. In the current study, we examined the magnitude of non-uptake of GERA testing among participants randomized to the intervention arm, and tested several hypotheses for why test uptake was not occurring. Our hypotheses and analyses were guided by the existing literature and the Preventive Health Model and Precaution Adoption Process Model behavioral models, which together informed the selection of measures and outcomes in the larger trial. Our first hypothesis was that lower knowledge (of genetics, gene-environment risks, and CRC) in the study population would be associated with lower uptake of the GERA test. We anticipated that modest to low levels of knowledge would be seen due to the novel experimental nature of GERA and the low objective risk of CRC in this average risk adult population contributing to low perceived relevance of the GERA test. Secondly, we hypothesized that AA race would also be associated with lower uptake of the GERA test. Despite the possible attractiveness of a combination gene-environment test to AAs and expressed interest and consent to be in the study, we believed that actual GERA testing uptake would be less than in white participants, similar to the low uptake of the testing among AAs who completed baseline surveys in research from the Multiplex initiative research [6,29,32] and Bloss *et al. *[33]. A third hypothesis was that perceived risk of cancer among participants would not be associated with GERA uptake, as we anticipated generally low levels of perceived risk in this cohort. Finally, we further hypothesized that race, with its previously documented associations to knowledge of genetics and genetic risk perception, would moderate the relationships of the other two predictors of interest to uptake of the GERA test. In line with the literature and our behavioral models, we included age, sex, educational attainment, and marital status as relevant covariates in our assessment of the hypothesized predictors [6-16,34,35].

## Materials and methods

### Data source

A detailed description of the GERA methods has been published [31]. In brief, eligible participants for the GERA study include average risk adults non-compliant with recommended CRC screening. Participants are drawn from family medicine and internal medicine practices affiliated with Jefferson Medical College. A software program screens the billing and scheduling databases of the participating medical practices for eligible patients. Initially only patients with a scheduled appointment were targeted for recruitment. Because of slow accrual, the investigative team expanded recruitment to patients without appointments. Potential participants are mailed a personalized invitation to participate. Telephone contact is made with eligible participants who do not opt out to obtain informed consent and to administer a baseline survey. All patients are given the opportunity to ask questions before the informed consent document is signed. Following completion of the survey, participants are randomly assigned 1:2 to a usual care/Control group or the Intervention group, usual care plus GERA (red blood cell folate level plus assessment of two germ-line SNPs in the methylenetetrahydrofolate reductase gene (*MTHFR*)). An appointment for the GERA blood draw is made, either in conjunction with or independent of a physician office visit. A second informed consent to have the blood draw and GERA risk testing is obtained at the testing appointment.

At the time of the baseline survey, participants were alerted that they could be invited to have a blood draw at the time of the decision counseling. At decision counseling, participants were presented with the GERA pamphlet and received education emphasizing how GERA information might inform individual decision making about CRC screening. The concept that GERA information is not a substitute for CRC screening, but rather a method to stratify risk, is emphasized. It was also emphasized that GERA would not provide a numerical magnitude of risk elevation, but would only gauge CRC risk as 'not elevated' or 'elevated'. Participants are allowed to take the GERA pamphlet home. The pamphlet contains a toll-free telephone number if the recipient has further questions.

### Study population

**Figure 1:**
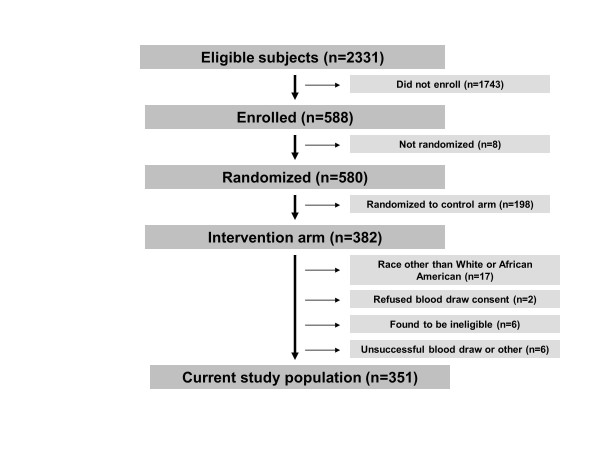
**Flowchart for study subjects drawn from the Gene Environment Risk Assessment (GERA) trial**.

A flow chart of the study participants is shown in Figure 1. At the time of the current analysis, 580 participants had enrolled in the trial and were randomized. The trial was recently closed after 748 participants signed consent and were randomized. Of the 580 participants analyzed here, 382 (65.9%) were randomized to the Intervention group. Seventeen of these participants (17/382) reported a race other than AA or white and were excluded from the current analysis. Of the remaining AA and white participants (*n *= 365), 2 individuals (0.5%) attended the testing visit but refused to sign informed consent for the blood draw. An additional 12 participants (3.3%) were excluded for study ineligibility (*n *= 6), unsuccessful blood draw (*n *= 2), or miscellaneous causes (*n *= 4). Thus, the sample size for this analysis includes 351 participants, representing 91.9% (351/382) individuals randomized to the Intervention who were eligible for the GERA test. A secondary analysis of patients accepting versus declining participation in the GERA trial found that participants were younger than non-participants (*P *< 0.001), but no differences by race or gender were seen.

Participants have multiple opportunities to complete the GERA risk testing, and several repeat attempts to schedule testing are made. Reasons for GERA testing non-completion include: 1) failure of the patient to attend a scheduled appointment for consent and blood draw; 2) inability of study staff to successfully schedule an appointment for blood draw and consent after previously obtaining a patient's consent to enroll in the study. The vast majority of patients who did not complete GERA testing either cancelled their testing visit on multiple occasions or simply did not show up, and did not respond to multiple attempts to reschedule.

### Conceptual model and study measures

Selection of measures for the baseline survey for the larger randomized GERA clinical trial is guided by two behavioral health models, the Preventive Health Model and the Precaution Adoption Process Model [31,36,37]. The former predicts that preventive health behaviors may be influenced by demographic factors, cognitive perceptions of the health threat, and perceived risk of the health threat [36,37]. In the current study, with influence from the existing literature of genetic susceptibility testing and predictive genetic testing, and our own research examining the association of knowledge to decisions about genetic testing [16], we elected to focus in particular on race as the primary demographic factor of interest, objective knowledge (of gene-environment risk of cancer, genetics, and colorectal cancer screening) as the primary cognitive factor, and perceived risk (here, comparative risk) as the predominant potential predictors within the model, and considered uptake of the GERA screening test as the target screening behavior.

**Figure 2:**
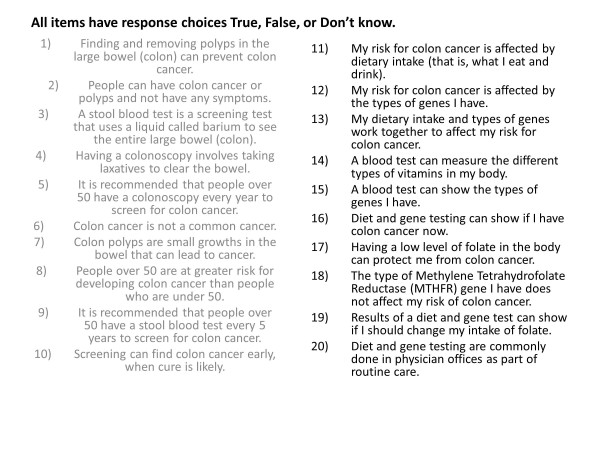
**Knowledge items from the baseline questionnaire of the Gene Environment Risk Assessment (GERA) trial**.

Age, race, gender, marital status, education, and knowledge are obtained from the baseline survey. This survey contains 20 face-valid true/false items developed by the investigative team assessing knowledge of colorectal cancer, genetics and gene-environmental risk for colorectal cancer (Figure 2). Additional baseline survey items assess perceived risk of CRC by a single item measured on a five-point Likert scale ('compared to other persons my age, I am at lower risk for colon cancer'), and items assessing marital status, gender, education, and age. Self-reported race was recorded via eight categories (American Indian or Alaskan Native, Asian, Black or African American, Native Hawaiian or other Pacific Islander, White, More than one race, Don't know, and Refused). Ethnicity was recorded as Hispanic or Latino, Not Hispanic or Latino, Don't know, or Refused. For this analysis, individuals reporting White or Black race are included, as these two groups represented 97% of study participants. A small number of individuals reporting Black or White race and Hispanic or Latino ethnicity are included in these analyses.

### Analyses

Age is treated as a continuous variable. Knowledge includes total knowledge items answered correctly (*n *= 20 items). Knowledge sub-scores are also reported for CRC risk factor and screening items (knowledge of CRC, *n *= 10) and for dietary and genetic risk factor items (knowledge of GERA, *n *= 10). GERA testing uptake is reported as the proportion of participants randomized to the GERA intervention that completed testing. Univariate associations between predictors and uptake are reported as odds ratios (OR) with 95% confidence intervals (CI). Multivariable logistic regression was used to examine the association of race, perceived risk, and knowledge to GERA testing uptake. Moderation by race was assessed by creating an interaction term and testing its significance in the multivariable model. All confidence intervals are reported at the 95% level, and alphas are reported as significant when < 0.05. All analyses were performed using STATA9SE.

The GERA study has been approved by the Institutional Review Boards of Fox Chase Cancer Center and Jefferson Medical College, and conforms to the Declaration of Helsinki.

## Results

### Characteristics of participants stratified by GERA uptake

Baseline characteristics of participants randomized to the GERA testing arm and considered for this analysis are presented in Table 1. The mean age among all participants was 60.1 years. Participants were more likely to be female (59.0%) and white (54.1%). Approximately one-third had a college degree (36.8%). Despite the study inclusion of low risk participants by personal and family history criteria, 151 (43.0%) participants estimated their risk of CRC to be higher than other persons their age. On the 20-item CRC knowledge assessment, median number of items correct among all participants was 12/20 (standard deviation 3.67).

**Table 1:** Characteristics of participants eligible to undergo GERA risk testing (*n *= 351)

	GERA risk testing uptake			
			
	Yes	No		
Subject characteristics	*n *= 249 (70.9)	*n *= 102 (29.1)	OR	95% CI
**Age: mean (SD)**	59.8 (7.2)	60.8 (8.1)	0.98	0.95-1.01
**Sex: n (%)**				
Female	148 (71.5)	59 (28.5)	1.07	0.67-1.70
Male	101 (70.1)	43 (29.9)	--	--
**Race^a^: n (%)**				
African American	96 (59.6)	65 (40.4)	0.36^b^	0.22-0.58
White	153 (80.5)	37 (19.5)	--	--
**Marital status: n (%)**				
Married	131 (77.1)	39 (22.9)	1.78^c^	1.11-2.84
Unmarried/other	118 (65.2)	63 (34.8)	--	--
**Education: n (%)**				
High school or less	69 (61.1)	44 (38.9)	Referent	Referent
Some college	72 (66.1)	37 (33.9)	1.24	0.72-2.15
College graduate or more	108 (83.7)	21 (16.3)	3.28^b^	1.80-5.98
**Perceived risk^d^: n (%)**				
Agee (perceived average risk)	76 (75.3)	25 (24.7)	Referent	Referent
Unsure	71 (71.7)	28 (28.3)	0.83	0.44-1.56
Disagree (perceived elevated risk)	102 (67.6)	49 (32.5)	0.68	0.39-1.21
**CRC knowledge mean score: median (SD)**				
All questions (20 items)	12 (3.53)	10 (3.73)	1.14^b^	1.07-1.21
Risk factors and screening (10 items)	7 (1.86)	6 (2.05)	1.33^b^	1.18-1.50
Diet and genetics (10 items)	5 (2.16)	5 (2.11)	1.14^c^	1.02-1.27

^a^Includes nine individuals of Hispanic ethnicity (eight AA, one White). ^b^*P *< 0.001. ^c^*P *= 0.02. ^d^'Compared with other persons my age, I am at lower risk for colon cancer'. CI, confidence interval; CRC, colorectal cancer; GERA, Gene Environment Risk Assessment; OR, odds ratio; SD, standard deviation; SE, standard error.

### Uptake of GERA risk testing

Among the 351 subjects eligible for GERA risk testing, 249/351 (70.9%) completed testing. Some variability in uptake by baseline demographic covariates was seen. Univariate associations are presented in Table 1. Subjects who completed GERA testing were more likely to be married (OR 1.78 (95% CI 1.11 to 2.84)) and to have a college degree (OR 3.28 (95% CI 1.80 to 5.98)) compared to those who did not complete testing. We also examined the relationships of the primary predictors we were examining to uptake of GERA risk testing by participants. Predictors included race, perceived risk, and knowledge. AA participants were significantly less likely to complete GERA risk testing compared to white participants (OR 0.36 (95% CI 0.22 to 0.58)). Total knowledge score was also associated with testing completion (OR 1.14 (95% CI 1.07 to 1.21)). There was no association seen between perceived risk for colon cancer and uptake of testing.

### Multivariable model

A multivariable logistic regression model including the three predictors (race, perceived risk, and knowledge) was next developed to further examine the relationships identified in the univariate analyses (Table 2). Covariates age, sex, marital status, and educational attainment were included in the model. In the complete model containing race, knowledge, and perceived risk, AA race was negatively associated with GERA testing completion (OR 0.51 (95% CI 0.29 to 0.87)), while increasing knowledge score was positively associated with completion (OR 1.09 (95% CI 1.01 to 1.18)). Interestingly, being married was also found to be significantly associated with uptake of testing in the model (OR 1.81 (95% CI 1.09 to 3.00)). The role of race as a moderator of the relationship between knowledge and uptake or perceived risk and uptake was assessed using interaction terms, but both terms were found to be non-significant.

**Table 2:** Multivariable models examining predictors of GERA risk testing uptake

	All participants	
	
Independent variable	Risk testing uptake: OR (95% CI)	*P*
**Knowledge**		
Total knowledge	1.09 (1.01-1.18)	0.03
**Race**		
White	Referent	Referent
African American	0.51 (0.29-0.87)	0.01
**Perceived risk**		
Below average	Referent	Referent
Unsure	0.92 (0.45-1.85)	0.80
Above average	0.78 (0.42-1.44)	0.43
**Education**		
High school or less	Referent	Referent
Some college	0.91 (0.50-1.65)	0.75
College graduate +	1.67 (0.83-3.36)	0.15
**Marital status**		
Unmarried/other	Referent	Referent
Married	1.81 (1.09-3.00)	0.02
**Interaction terms**		
Race*knowledge	--	NS
Race*comparative risk	--	NS

Results adjusted for age and sex. CI, confidence interval; GERA, gene environment risk assessment; NS, not significant.

## Discussion

We found incomplete uptake/completion of an experimental GERA test of modest predictive utility despite extensive efforts to complete risk testing in patients randomized to the intervention group in the GERA trial. AA race and individuals with lower knowledge (of CRC, screening, and gene-environment risks and testing) were less likely to complete GERA risk testing in univariate and multivariable logistic regression analyses, while perceived cancer risk was not associated with uptake. Knowledge and race were each significant predictors of uptake of testing in the multivariable model. Race was not found to moderate the association of knowledge to uptake or perceived risk to uptake.

These findings support our first two hypotheses, that knowledge and race are independent predictors of uptake of the GERA test, and are consistent with the published literature. The importance of objective knowledge of a moderate risk gene-environment susceptibility test in moderating uptake in an average risk population is notable because this association is adjusted for education level and is identified in a consented clinical trial population enriched in AAs. This suggests that average risk patients may be more skeptical of an experimental genetic test about which they have limited understanding and exposure [9,12,15], or in the case of the GERA population, reluctant to have a non-standard test that may serve to remind them of their non-compliance with CRC screening [9,14]. Lower knowledge may also have contributed to participants questioning the relevance of the test for them, given their minimal family history and given possible perceptions of low personal risk of being folate deficient [6,8,9]. The significance of our findings associating race with low uptake of GERA are also interesting. While a negative association between AA race and uptake of predictive genetic testing has been documented several times in the literature, it has, to our knowledge, not been observed in an objectively low-risk population offered a dual gene environment susceptibility feedback assessment. Though research from White *et al. *[13] has suggested that research that considers both genetic and exposure-related causes of disease to be more acceptable to AAs, AA participants may have been less likely to complete GERA testing because of reasons not measured in the current analysis. Socioeconomic barriers, higher prevalence of fatalism toward colon cancer risk or distrust of the medical system and clinical trial research [34], or a higher likelihood of having already decided to get CRC screening, may all contribute to reducing the perceived relevance of the GERA test in the study population [8].

Our third hypothesis was also supported by the analyses, but should be interpreted with more caution. We anticipated low perceived risk in the GERA cohort based on the study selection criteria, and thus did not predict an association with GERA uptake in line with the Preventive Health Model. We were therefore surprised to find that 43% of the total study population reported their CRC risk to be higher than average. Nonetheless, perceived risk was not significant in the final model, and no interaction between perceived risk and race was identified. Elevated perceived risk here may be related to study selection bias, with more concerned individuals being more likely to consent to the research, or to changing perceptions of risk among participants within the context of the study. Nonetheless, that perceived risk was not significant in the model suggests that even those individuals who perceived higher risk may not have seen the GERA test as valuable to them, perhaps because they already recognized that they were higher risk, or because the GERA test did not quantify risk, but only reported risk qualitatively as elevated or not elevated. Our final hypothesis, that race may moderate both the relationships of knowledge and perceived risk to uptake, was disproven in our analyses. This both suggests that the mitigating effects of low knowledge on uptake of the GERA test were independent of race, and that race-specific variability in perceived risk was not influential in these findings.

The strength of the positive association of testing uptake with being married was unexpected. Marital status has previously been shown to predict prostate cancer screening [38], but most studies of CRC screening have not shown an association [39]. Being married may be associated with improved adherence to health behaviors such as cancer screening [40], and may indirectly be associated with follow-through for the GERA test by a consented patient. In line with the Precaution Adoption Process Model, spousal support may have served to motivate conflicted participants to complete the GERA testing in the setting of questionable health relevance or benefit to the study test. The relative impact of marital status may also be particularly enhanced by the genetic nature of the GERA test. Spouses may be particularly motivated to understand genetic risks in their partners and thus to motivate completion of study participation because of the assumed far-reaching implications of even a small increased genetic risk of colon cancer. Motivations may include to better mitigate high risks in their spouse, appropriately plan for possible future health problems and needs, and to protect children and other loved ones from similar health-related problems.

Several studies incorporating genetic susceptibility feedback testing have reported detailed information on uptake, but only a handful have commented on predictors of patient utilization of SNP-based biomarker testing for disease risk. A study examining uptake of the *GSTM1 *biomarker via an online portal among patients recruited in tandem through a smoking cessation trial found significantly lower completion of testing (38%) relative to reported interest in the available testing (63%) [11]. When predictors of completion of testing were examined, uptake was significantly associated with age (highest for 34 to 45 years; *P *= 0.04), awareness of genetic testing (*P *= 0.04), greater motivation to quit smoking (*P *= 0.03), and with internet access (*P *= 0.02). Updated data from Hensley-Alford *et al. *[32] reported as part of the ongoing Multiplex Initiative demonstrate AA race as a significant negative predictor of uptake of a novel SNP-based risk assessment panel. In particular, AAs were overall more indecisive about genetic testing than whites and were less likely to test (30% uptake among AAs vs 55% uptake among whites, *P *< 0.0001). AAs from lower education neighborhoods were least likely to complete the Multiplex testing. The Multiplex group has previously reported [6] several demographic characteristics associated with completion of the baseline survey (*n *= 1,959; 37% college graduate, 46% male, 37% white). Bloss *et al. *recently reported an association of race to completion of a direct-to-consumer SNP-based genomic disease risk profile, with non-Whites being less likely to complete testing and follow-up [33].

Individuals have been shown to express moderate to high interest in genetic testing despite low knowledge and understanding, but studies of testing uptake demonstrate markedly lower rates of testing completion among the underserved [21,26,41]. In particular, lower rates of genetic testing uptake have been identified among AAs [21,26,27]. Putative societal and socioeconomic factors related to uptake include cost, insurance barriers, and access to medical services for testing and counseling. Recent literature from White *et al. *[13] has suggested that AA patients may find approaches to disease risk that focus only on genetics less appealing. Persistent cultural barriers also include racial/ethnically founded differences in fatalism or fears of genetic discrimination [21,27,28,42] even when education levels are high [43]. Finally, research from Armstrong *et al. *has highlighted apparent greater concerns about honesty in the US healthcare system among AAs [34].

The average risk public lacks knowledge of genetics and genetic testing [17-20]. The impact of low knowledge about CRC, screening, or gene-environment risks on uptake of an experimental gene-environment genetic susceptibility test may be mediated by understanding (of testing terms or mechanisms), or perceived relevance of testing. Whether parallels of testing experience in the GERA study can be drawn from the predictive genetic testing literature is uncertain. When predictive genetic testing has been studied, greater objective knowledge has generally predicted testing uptake among individuals at increased objective and perceived risk, while more knowledgeable persons at lower risk show less interest in testing [44], presumably because they are more aware of their lower risk status [24,44]. Underserved and minority populations have been found to have notably lower objective knowledge of genetic testing [17,18,21-25,42]. It is possible that in the setting of relatively low knowledge about genetics or experience with an experimental genetic test like GERA, perceived risk of cancer may be less strongly associated with interest in predictive genetic testing [23,45,46].

The strengths of the current study include the size of the study sample, the large number of AA participants in the study, and the non-hypothetical nature of the genetic susceptibility testing offered to this diverse cohort. The unique nature of the GERA testing, incorporating both genetic markers of risk and a dietary marker, also permits the exploration of how the public may accept purely genetic susceptibility tests versus those that offer exposure-related determinants of disease.

Among the limitations to this analysis is the inability to further understand how individuals who opted to not complete testing made this decision. Although unlikely, it is also conceivable that a small number of individuals who did not complete testing will still attempt to complete testing. The small number of non-white non-AA participants has made analyses of testing uptake for other underserved or racial/ethnic groups impossible. Income data were also not collected, limiting our ability to incorporate this relevant factor into our multivariable logistic models. Finally, because GERA testing is not standard of care, and was offered in the context of a clinical trial, it remains uncertain whether our study findings would predict the behavior of patients faced with a recommended or standard genetic susceptibility test in a real-world setting.

## Conclusions

Multiple factors influence uptake of a moderate risk gene-environment CRC susceptibility test among AA and white patients participating in a clinical trial setting, including objective risk status of the study population, objective knowledge, race, and marital status. In particular, establishing relevance of a gene-environment test for disease risk may be challenging in an objectively low-risk, minority rich population with limited knowledge of the novel intervention test. Recruiting and supporting clinical study participation and completion in a genetic susceptibility study among average risk AAs will also continue to be a challenge. Future studies incorporating genetic susceptibility biomarkers may consider expanded efforts to tailor pre-testing education to particularly difficult to reach groups like AA men [47] and to better engage AA clinical trial participants to improve study completion and follow-up. Public campaigns to improve knowledge of CRC screening, risk factors, and genetics could enable patients to make more informed genetic testing choices [44,46]. Knowledgeable adults faced with multiple health risks and financial concerns may conclude that a particular test, particularly one that conveys only a modest increase in risk, is not sufficiently important to warrant its uptake/completion.

## Abbreviations

AA: African American; CI: confidence interval; CRC: colorectal cancer; GERA: gene-environment risk assessment; OR: odds ratio; SNP: single nucleotide polymorphism.

## Competing interests

The authors declare that they have no competing interests.

## Authors' contributions

MJH: **c**onception, design, analysis, interpretation, manuscript drafting. SLM: conception, design, analysis, interpretation, editing. REM: **c**onception design, analysis, interpretation, editing. EMK: acquisition of data. AMB: acquisition and analysis of data. DSW: conception, design, analysis, interpretation, editing. All authors read and approved the final manuscript.
